# Antimicrobial Activity of *Eucalyptus globulus*, *Azadirachta indica*, *Glycyrrhiza glabra*, *Rheum palmatum* Extracts and Rhein against *Porphyromonas gingivalis*

**DOI:** 10.3390/antibiotics11020186

**Published:** 2022-01-31

**Authors:** Lena Katharina Müller-Heupt, Nina Vierengel, Jonathan Groß, Till Opatz, James Deschner, Friederike D. von Loewenich

**Affiliations:** 1 Department of Oral and Maxillofacial Surgery, University Medical Center Mainz, Augustusplatz 2, D-55131 Mainz, Germany; 2 Department of Chemistry, Johannes Gutenberg-University, Duesbergweg 10–14, D-55128 Mainz, Germany; vierengel@uni-mainz.de (N.V.); jgross03@uni-mainz.de (J.G.); opatz@uni-mainz.de (T.O.); 3 Department of Periodontology and Operative Dentistry, University Medical Center Mainz, Augustusplatz 2, D-55131 Mainz, Germany; james.deschner@unimedizin-mainz.de; 4 Department of Medical Microbiology and Hygiene, University of Mainz, Obere Zahlbacherstr. 67, D-55131 Mainz, Germany; friederike.loewenich@unimedizin-mainz.de

**Keywords:** *Azadirachta indica*, broth microdilution, *Glycyrrhiza glabra*, plant extracts, *Porphyromonas gingivalis*, rhein, *Rheum palmatum*

## Abstract

Novel plant-derived antimicrobials are of interest in dentistry, especially in the treatment of periodontitis, since the use of established substances is associated with side effects and concerns of antimicrobial resistance have been raised. Thus, the present study was performed to quantify the antimicrobial efficacy of crude plant extracts against *Porphyromonas gingivalis*, a pathogen associated with periodontitis. The minimal inhibitory concentrations (MICs) of *Eucalyptus globulus* leaf, *Azadirachta indica* leaf, *Glycyrrhiza glabra* root and *Rheum palmatum* root extracts were determined by broth microdilution for *P. gingivalis* ATCC 33277 according to CLSI (Clinical and Laboratory Standards Institute). The MICs for the *E. globulus*, *A. indica* and *G. glabra* extracts ranged from 64 mg/L to 1024 mg/L. The lowest MIC was determined for an ethanolic *R. palmatum* extract with 4 mg/L. The MIC for the anthraquinone rhein was also measured, as the antimicrobial activity of *P. palmatum* root extracts can be partially traced back to rhein. Rhein showed a remarkably low MIC of 0.125 mg/L. However, the major compounds of the *R. palmatum* root extract were not further separated and purified. In conclusion, *R. palmatum* root extracts should be further studied for the treatment of periodontitis.

## 1. Introduction

According to the Global Burden of Disease Study, severe periodontitis had a prevalence of nearly 10% of the global population in 2016 [1]. It is a chronic inflammatory disease of tooth-surrounding tissues causing soft tissue destruction and alveolar bone loss and, thus, may ultimately lead to tooth loss [2]. Furthermore, it has been associated with an increased risk of all-cause mortality and several diseases such as cardiovascular disease, cancer, diabetes mellitus, psoriasis and rheumatoid arthritis [3,4,5,6,7].

The initiation and progression of chronic periodontitis is a multifactorial process that is driven by *Porphyromonas gingivalis,* an obligate anaerobe Gram-negative rod that is part of the human oral flora [8]. Alongside being a pathogen in periodontitis, *P. gingivalis* may induce systemic inflammation and has been correlated with the development of systemic disorders such as cardiovascular and rheumatic disease [9].

The gold standard in the therapy of periodontitis is the mechanical subgingival instrumentation [10], although due to the complex anatomy of periodontal pockets and teeth, this procedure is unable to fully eradicate bacterial biofilms. The use of systemic antimicrobials is controversial and limited to aggressive and severe forms of periodontitis [11]. Thus, the adjuvant use of antiseptics such as chlorhexidine (CHX) mouthwash is a common part of the treatment concept for chronic periodontitis [12]. The main side effect of CHX is tooth staining [13]. Additionally, although not yet a clinical problem in dentistry, concerns about the development of bacterial resistance against CHX have been raised [14]. Therefore, there is increasing interest in novel antimicrobial agents for the local long-term treatment of chronic periodontitis. Plants are of high interest to discover new antibacterial agents, since they are naturally exposed to microbial infections and thus have developed various antimicrobial defense mechanisms. Various plant extracts have been used for centuries in traditional medicine and there is some clinical evidence for their potential to control periodontitis progression [15,16]. The most commonly discussed medicinal plants for use in dentistry are *Eucalyptus globulus, Azadirachta indica, Glycyrrhiza glabra* and *Rheum palmatum.*

*E. globulus,* known as fever tree, is a member of the Myrtaceae family. A chewing gum containing 0.6% *E. globulus* ethanolic leaf extract has shown statistically significant positive effects on various outcome parameters of gingivitis in a small double-blind randomized clinical trial [17].

*A. indica* (neem) belongs to the Meliaceae family and is a traditional medicinal herb used in India to maintain oral health [18,19]. Clinical trials with a low number of 20 subjects used either an *A. indica* extract-containing gel or non-absorbable neem oil chips as an adjunct to subgingival instrumentation. Both gel and chips led to further improvement on pocket probing depth and attachment level compared to subgingival instrumentation only [20,21]. Further, a systematic review found no statistical differences in treatment outcomes between mouth rinses containing *A. indica* extracts or CHX, although the quality of the randomized controlled trials included was low [22].

*G. glabra* (licorice) is a member of the Fabaceae family and one of the oldest plants of ayurvedic medicine [23]. The bioactive compounds of its roots had anti-inflammatory properties in vitro, although evidence for clinical efficacy in periodontitis is lacking so far [24].

*R. palmatum* (rhubarb) is a member of the Polygonaceae family. Its root extract has been used as “Dahuang” in traditional Chinese medicine for centuries [25]. However, convincing evidence for its clinical activity against periodontitis has not yet been demonstrated.

Apart from their use in traditional medicine, at least some evidence for clinical efficacy in periodontitis or in vitro activity exists for most of the medicinal plants mentioned above. Given the fact that *P. gingivalis* is a major periodontal pathogen [8], we chose to investigate the antimicrobial activity of *E. globulus, A. indica, G. glabra* and *R. palmatum* extracts against *P. gingivalis*. The interpretation of studies reporting the antimicrobial activity of plant extracts is hindered by the fact that often insufficient techniques, such as disk diffusion, were used [26]. Therefore, we chose to compare the antimicrobial activity of extracts of the four plants against *P. gingivalis* using broth microdilution (BMD), as it represents the reference method of antimicrobial susceptibility testing [27].

To the best of our knowledge, no direct comparison of the antimicrobial activity of these extracts against *P. gingivalis* has been undertaken so far. The highest antimicrobial activity was found here for *R. palmatum* root extracts. Therefore, the anthraquinone rhein, a constituent of *R. palmatum* with known antimicrobial activity [28], was included in the analysis.

## 2. Materials and Methods

### 2.1. Plant Extracts

200 g of dried powder of pharmaceutical-grade *E. globulus* leaves (Bristol Botanicals, Bristol, UK), 200 g of dried powder of food-grade *A. indica* leaves (Vitafoodz, Waren, Germany) and 200 g of dried powder of pharmaceutical grade *G. glabra* roots (Bristol Botanicals) were extracted using either 600 mL 70% aqueous ethanol (Carl Roth, Karlsruhe, Germany) or 600 mL acetone (Carl Roth) for 24 h under continuous stirring. Insolvable parts were taken off using a 0.22 µm Stericup vacuum filtration system (Merck Millipore, Billerica, MA, USA). The extractant was then removed under reduced pressure at 40 °C using a rotary evaporator (Rotavapor R-210, Büchi, Essen, Germany). The soluble fraction was weighed and dissolved in DMSO (Carl Roth), achieving a stock concentration of 204.8 g/L. Aliquots were stored at −80 °C and diluted in DMSO to a final concentration of 20.48 g/L prior to use. Dried *R. palmatum* root extract (2.048 g, Paninkret, Pinneberg, Germany) was solved according to the instructions of the manufacturer in 100 mL 50% aqueous ethanol (Carl Roth), achieving a stock concentration of 20.48 g/L. The stock solution was stored at 4 °C. 2.048 g rhein (Sigma Aldrich, Darmstadt, Germany) was dissolved in 100 mL 0.1 M NaOH_(aq.)_ (Carl Roth), achieving a stock concentration of 20.48 g/L. The stock solution was stored at room temperature protected from daylight.

### 2.2. Broth Microdilution (BMD)

*P. gingivalis* (DSM 20709, ATCC 33277) was obtained from the German Collection of Microorganisms and Cell Cultures (DSMZ). The stock was stored in liquid nitrogen using the cryobank system from Mast Diagnostika (Reinfeld, Germany). A working aliquot was held at −70 °C and used for sub-culturing the strain each week at 37 °C on Schaedler agar (Becton Dickinson, Heidelberg, Germany) under anaerobic conditions (90% N_2_, 10% CO_2_, 10% H_2_). A fresh 48 h-old isolation of *P. gingivalis* was used for the inoculation of BMD. Anaerobiosis was controlled by indicators. All susceptibility tests were performed according to CLSI (Clinical and Laboratory Standards Institute) [27] with the exception that Wilkins–Chalgren broth (Merlin, Bornheim-Hersel, Germany) was used instead of supplemented Brucella broth. The minimal inhibitory concentration (MIC) of each test substance was determined as the lowest concentration that prevented the visible growth of *P. gingivalis* ATCC 33277 after 48 h of incubation and was expressed in mg/L. An inoculum with turbidity equal to 0.5 McFarland was used. Serial dilutions revealed that such a suspension contained approximately 1 × 10^7^ *P. gingivalis* ATCC 33277 colony-forming units (CFU)/mL. The bacterial colonies were suspended in 1.5 mL sterile 0.9% NaCl_(aq.)_ solution until a turbidity of 0.5 McFarland was reached. The turbidity was controlled using a photometric device (DensiCheck Plus, bioMerieux, Nürtingen, Germany). The bacterial suspension was then diluted 1:10 in Wilkins–Chalgren broth. 50 µL were used to inoculate the wells of a polypropylene microdilution tray (Greiner, Frickenhausen, Germany). 1 mL of the test substance stock (20.48 g/L) was diluted in 9 mL Wilkins–Chalgren broth. 50 µL was mixed with 50 µL of the bacterial suspension, reaching a final test substance concentration of 1024 mg/L. The bacterial inoculum was equivalent to 5 × 10^5^ CFU contained in a total test volume of 100 µL. Lower test substance concentrations were reached by serial dilution. Each test substance concentration was tested in quadruples or octuples depending on the respective experiment. Each experiment was repeated five times. Control rows contained 100 µL of Wilkens–Chalgren broth alone, 100 µL of the bacterial inoculum alone as growth control, or 100 µL of the test substance alone. Microdilution trays were covered with a perforated plastic foil (reference number M/B3-002-040, Sifin Diagnostics, Berlin, Germany) and incubated for 48 h in an anaerobic jar under anaerobic conditions (see above). Selected wells were sub-cultured on Schaedler agar as purity controls. The growth of bacteria other than *P. gingivalis* was not observed. The control experiments were performed with the respective solvent (DMSO, 50% aqueous ethanol, 0.1 M NaOH_(aq.)_) alone and repeated five times. Growth inhibition due to the solvent alone was not detected.

### 2.3. Quality Control

The performance of the anaerobic test system was monitored by the concomitant use of the control strains *Bacteroides fragilis* ATCC 25285 and *B. thetaiotaomicron* ATCC2 9741 and the MICRONAUT-S anaerobes MIC plates (reference number E1-085-040, Merlin). *B. fragilis* and *B. thetaiotaomicron* were cultured on Columbia blood agar (Becton Dickinson) at 37 °C under an anaerobic atmosphere for 24 h (*B. fragilis*) or 48 h (*B. thetaiotaomicron*) and used to inoculate MICRONAUT-S anaerobes microtiter plates according to the instructions of the manufacturer. The MICs were determined after an incubation of 24 h (*B. fragilis*) or 48 h (*B. thetaiotaomicron*) under anaerobic conditions using a Tecan Sunrise microplate reader (Tecan, Crailsheim, Germany) and the MCN6 software (Merlin). Quality control ranges for the respective antimicrobials were applied according to the instructions of the manufacturer (Supplementary Materials Table S1). The quality controls were performed weekly and quality control ranges were always reached.

### 2.4. HPLC (High-Performance Liquid Chromatography) Analysis

The analysis of the crude extracts was performed on an Agilent Infinity II 1260 system with a diode array detector. An ACE C18-PFP column (150 mm × 4.6 mm, 3 μm, 40 °C) was applied as stationary phase. A gradient mixture of acetonitrile and water (containing 0.1% formic acid) with a constant flow rate of 1.0 mL/min was used as an eluent with the following linear gradient elution program: 0 min: 99% H_2_O and 1% MeCN, 30 min: 5% H_2_O and 95% MeCN, 40 min: 5% H_2_O and 95% MeCN. For HPLC analysis, the material was dissolved in a 1:1 (*v/v*) MeCN/H_2_O solution arriving at a concentration of 1.02 mg/mL. The resulting samples were filtered over a Macherey–Nagel syringe filter with a PTFE membrane (0.2 µm pore size) prior to injection with an injection volume of 3 μL.

## 3. Results

### 3.1. BMD

The median MICs of *E. globulus*, *A. indica*, *G. glabra* and *R. palmatum* extracts for *P. gingivalis* ATCC 33277 from five independent experiments are given in Table 1. The MICs of *E. globulus* leaf, *A. indica leaf* and *G. glabra* root extracts were lower when acetone was used as extractant compared to 70% aqueous ethanol (Table 1). This was most striking for the *G. glabra* root extracts where the median MIC of the 70%-ethanolic extract had a 16-fold higher MIC than the respective acetone extract (1024 mg/L versus 64 mg/L). The lowest MIC of all plant extracts (4 mg/L) was found for the *R. palmatum* extract dissolved in 50% aqueous ethanol.

**Figure 1:**
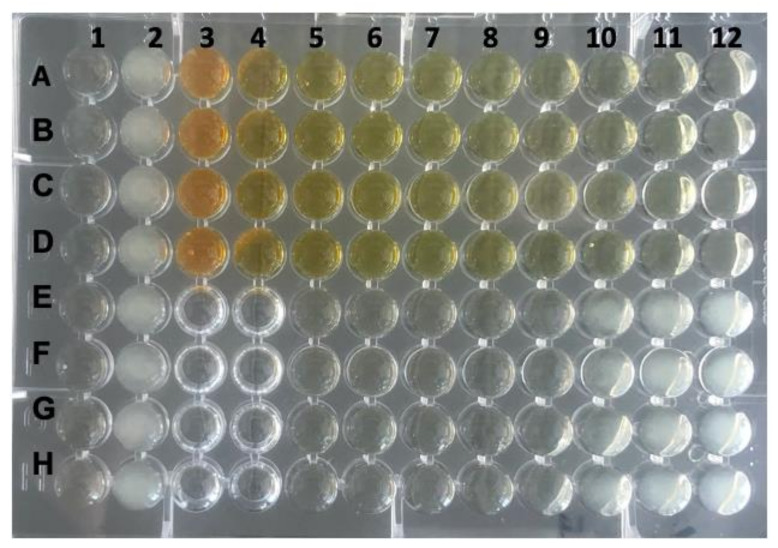
Determination of the MIC of rhein for *P. gingivalis* strain ATCC 33277. Column 1 served as negative control (Wilkins–Chalgren broth only), column 2 contained the growth control (*P. gingivalis* only) and column 3 wells A–D contained the test substance alone (rhein at a concentration of 2048 mg/L). A serial dilution of rhein was performed in quadruples, starting in column 4 wells A–D (concentration of 1024 mg/L) to column 12 wells A–D. The last row with prevented visible growth was column 9 wells E–H (MIC = 0.125 mg/L). Columns 3 and 4 wells E–H were left empty.

For *R. palmatum* root extract, it is known that its antimicrobial activity against *P. gingivalis* can be traced back partially to the anthraquinone rhein [29]. Therefore, the MIC of rhein dissolved in 0.1 M NaOH_(__aq.)_ for *P. gingivalis* ATCC 33277 was determined. The median MIC of rhein from five independent experiments was 0.125 mg/L (Table 1, Figure 1).

The solvents used (DMSO, 50% aqueous ethanol and 0.1 M NaOH_(aq.)_ showed no significant antimicrobial activity with MICs above 1024 mg/L (Table 2).

All extracts were further characterized by HPLC–MS. This was of special interest for the *R. palmatum* extract with regard to its rhein content and, considering that the oral enzymes are able to cleave glycosidic bonds, also its rhein glucoside content [30,31].

### 3.2. HPLC

**Figure 2:**
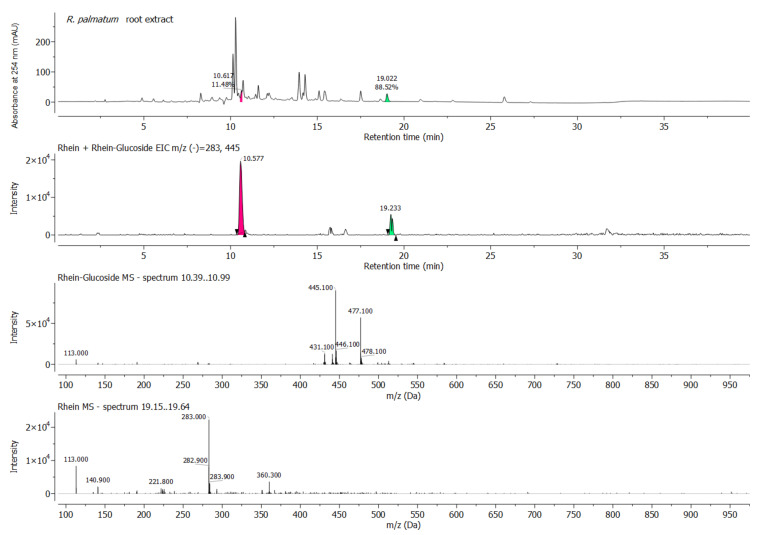
HPLC chromatogram (monitored at 254 nm), respective total ion current (TIC) ESI–MS (–) and extracted ESI–MS (–) spectrum of R. palmatum root sample at 10.39–10.99 min (pink, rhein-glucoside) and 19.21–19.34 min (green, rhein).

HPLC with diode array detection was performed to reveal the chemical fingerprints of the plant extracts tested herein. This will allow the comparison with upcoming samples of the same plant species in the future. As mentioned above, the acetone extracts of *E. globulus* leaves, *A. indica* leaves and *G. glabra* roots had lower MICs than the respective ethanol extracts. When comparing the chromatograms of the ethanol and acetone extracts, it is evident that the chemical complexity of the acetone samples is generally higher than that of the corresponding ethanol samples, which can be deduced from the number of peaks (Supplementary Materials Figures S1–S3, Tables S2–S7). In addition, it can be observed that more peaks with higher retention times appear in the acetone samples than in the peaks with lower retention times, with the former generally being attributed to more lipophilic substances (e.g., essential oils). However, a detailed component analysis was not performed. The chromatogram and the peak list of the rhubarb extract are shown in Figure 2 and Table S8.

In the instance of the *R. palmatum* root extract, it was suspected that its antimicrobial activity against *P. gingivalis* was partially derived from the presence of the anthraquinone rhein [29] and possibly its glycosylated form as well. This prompted us to further analyze the extract for its rhein content, for which purpose a sample of pure rhein was prepared and analyzed via HPLC. By comparing the retention time of rhein with the signals observed for the *R. palmatum* extract, it was noted that rhein was indeed present in the rhubarb sample, but in a relatively low concentration (Figure 2 and Figure S4, Tables S8 and S9). The comparison of the UV signal levels between the rhubarb root and the pure rhein sample indicated a rhein concentration of 5% in the *R. palmatum* extract. This nicely correlated with the BMD results, where the MIC ratio of the *R.* *palmatum* extract (4 mg/L) and rhein (0.125 mg/L) was 32. However, the major compounds of the *R. palmatum* root extract were not further separated and purified.

An extracted ion chromatogram (EIC) of rhein glucoside (m/z [M − H]^−^ = 445.1) and rhein (m/z [M − H]^−^ = 283.0) confirmed the presence of both forms (Figure 2). The m/z values of other known *R. palmatum* anthraquinones were also detected, but could not be individually assigned.

## 4. Discussion

This study was designed to evaluate plant extracts regarding their antimicrobial activity against *P. gingivalis* in vitro. BMD according to CLSI was chosen as the method, since it is standardized and therefore allows the comparison of the antimicrobial activity against *P.gingivalis* between the extracts tested. Many previous in vitro studies performed with *E. globulus, A. indica, G. glabra* or *R. palmatum* extracts do not allow a direct comparison between their antimicrobial activity due to the variety of methods used and the parameters applied, such as solvents, inocula, culture media and incubation periods. A common method used in other studies is disk diffusion. Due to their non-polarity, the compounds of plant extracts with the highest antimicrobial activity do not diffuse well in agar-based media. Therefore, disk diffusion is not appropriate to study the antimicrobial activity of plant extracts [26].

The antimicrobial activity of *Eucalyptus* essential oil or an *E. globulus* ethanolic leaf extract against *P. gingivalis* has been shown using disk diffusion [32,33]. However, the inhibition zones reported cannot be compared due to the reasons discussed above. In a study performed by Nagata et al., the growth of *P. gingivalis* ATCC 33277 was inhibited by macrocarpals A or B purified from a 60% ethanolic *E. globulus* leaf extract at a concentration of 1 mg/L [34]. This is much lower than in our study, as we measured a MIC of 128 mg/L for the acetone extract and of 256 mg/L for the ethanol extract, but it is reasonable that purified compounds exhibit stronger antimicrobial activities than crude extracts.

An ethanolic *A. indica* leaf extract showed a MIC of 500 mg/L against *P. gingivalis* ATCC 33277 in a previous study [35], which is comparable to our results of 1024 mg/L for the ethanol and 256 mg/L for the acetone extract.

A MIC of 62.5 mg/L of a 95% ethanol *G. glabra* root extract was reported. for *P. gingivalis* ATCC 33277 [36]. This matches the MIC of 64 mg/L we measured for the acetone extract, although our 70% ethanol extract showed a higher MIC of 1024 mg/L. This might be explained by the higher ethanol concentration used in the study mentioned above.

An 100% ethanolic extract of *R. palmatum* root showed a MIC of 500 mg/L for *P. gingivalis* ATCC 33277 in a previous study [29]. In our study, a 80%/50% *R. palmatum* root extract had the highest antimicrobial activity of all the extracts tested, with a MIC of 4 mg/L. The difference might be partially explained by the higher inoculum of 0.2 OD_660_ used by Liao et al. [29].

It was suspected that the antimicrobial activity of *R. palmatum* root extracts against *P. gingivalis* was partially derived from the presence of the anthraquinone rhein [29]. In a study by Azelmat et al., rhein showed a MIC of 2.5 mg/L for *P. gingivalis* ATCC 33277 [28]. In our study, we measured a MIC of 0.125 mg/L for rhein, which again can be partially explained by the higher inoculum of 0.2 OD_660_ used by Azelmat et al. [28].

A study investigating the antimicrobial activity of *R. palmatum* root extract demonstrated that anthraquinones others than rhein, such as emodin, aloe-emodin, physcion and chrysophanol, inhibited the growth of *Salmonella* Typhimurium [37]. Rhein had the lowest MIC against *S.* Typhimurium. In our study, the comparison of the UV signal levels between the rhubarb root and the pure rhein sample indicated a rhein concentration of 5% in the *R. palmatum* extract. This nicely correlated with the BMD results, where the MIC ratio of the *R. palmatum* extract (4 mg/L) and rhein (0.125 mg/L) was 32. Therefore, we hypothesize that in our setting, rhein might have been responsible for a relevant part of the antimicrobial activity. However, the major compounds of the *R. palmatum* root extract were not further separated and purified.

In a study investigating 56 clinical isolates of *P. gingivalis*, the MIC_90_ for amoxicillin/clavulanic acid was 0.5/0.25 mg/L, for clindamycin 0.25 m/L and for metronidazole 2 mg/L [38], which is interpreted as susceptible for systemic use according to CLSI 2021 [39]. Therefore, the local application of *R. palmatum* root extract, for which we determined a MIC of 4 mg/L, might be promising in inhibiting the growth of *P. gingivalis* in vivo. However, when treating periodontitis, it must be considered that the antimicrobial effect on the bacteria in biofilms cannot be necessarily inferred from the growth inhibitory effect on planktonic cells. Further, periodontitis is a multifactorial process that is not caused by *P. gingivalis* alone [8]. Given the potential toxicity and carcinogenicity of *R. palmatum* [40], local tolerance of *R. palmatum* root extracts has to be demonstrated. Further, their clinical efficacy remains to be shown before they could become an alternative to CHX. At least, the in vitro MIC of CHX for *P. gingivalis* ATCC 33277 was reported to be 4 mg/L [41], which is the same as for the *R. palmatum* root extract tested in our study.

## 5. Conclusions

Extracts of *E. globulus*, *A. indica* (neem), *G. glabra* (licorice), *R. palmatum* (rhubarb) and rhein showed antimicrobial activity against *P. gingivalis*, making them promising candidates for further study in the treatment of periodontitis. The lowest MIC of all plant extracts was found for the *R. palmatum* extract (4 mg/L) and for the anthraquinone rhein (0.125 mg/L). The *R. palmatum* extract had a rhein concentration of 5%. The MIC ratio of both of them was 32. Therefore, we hypothesize rhein might have been responsible for a relevant part of the antimicrobial activity. However, the major compounds of the *R. palmatum* root extract were not further separated and purified.

## Figures and Tables

**Table 1:** MICs of the *E. globulus*, *A. indica*, *G. glabra*, *R. palmatum* extracts and rhein for *P. gingivalis* ATCC 33277. The median MIC from five independent experiments is shown.

Plant and Plant Part	Extractant	Solvent	MIC (mg/L)
*E. globulus* leaf	Acetone	DMSO	128
*E. globulus* leaf	70% Ethanol	DMSO	256
*A. indica* leaf	Acetone	DMSO	256
*A. indica* leaf	70% Ethanol	DMSO	1024
*G. glabra* root	Acetone	DMSO	64
*G. glabra* root	70% Ethanol	DMSO	1024
*R. palmatum* root	80% Ethanol/50% Ethanol *	50% Ethanol	4
Rhein	n/a **_._	0.1 M NaOH_(aq.)_	0.125

Plant part, extractant and solvent are given in the respective columns. n/a = not applicable, * extracted half and half with 80% and 50% ethanol by the manufacturer, ** pure substance.

**Table 2:** MICs of the solvent controls DMSO, 50% aqueous ethanol and 0.1 M NaOH_(aq__.)_ Five independent experiments yielded the same MIC of >1024 mg/L for all three solvents used.

Solvent	MIC (mg/L)
DMSO	>1024
50% Ethanol	>1024
0.1 M NaOH_(aq.)_	>1024

## Data Availability

Data are contained in this manuscript or supplementary materials.

## Supplementary Materials

The following are available online at https://www.mdpi.com/article/10.3390/antibiotics11020186/s1. Figure S1: HPLC chromatograms (monitored at 254 nm) of *E. globulus* leaf acetone and 70% ethanol extracts. Figure S2: HPLC chromatograms (monitored at 254 nm) of *A. indica* leaf acetone and 70% ethanol extracts. Figure S3: HPLC chromatograms (monitored at 254 nm) of *G. glabra root* acetone and 70% ethanol extracts. Figure S4: HPLC chromatogram (monitored at 254 nm), respective total ion current (TIC) ESI–MS (–) and extracted ESI–MS (–) spectrum at 19.21–19.37 min (green, rhein) of a commercial rhein sample (Sigma Aldrich). Table S1: Quality control ranges for *B. fragilis* and *B. thetaiotaomicron*. Table S2: Peak list of the *E. globulus* leaf acetone extract chromatogram at 254 nm with a minimum area threshold of 0.5%. Table S3: Peak list of the *E. globulus* leaf 70% ethanol extract chromatogram at 254 nm with a minimum area threshold of 0.5%. Table S4: Peak list the of the *A. indica* leaf acetone extract chromatogram at 254 nm with a minimum area threshold of 0.5%. Table S5: Peak list of the *A. indica* leaf 70% ethanol extract chromatogram at 254 nm with a minimum area threshold of 0.5%. Table S6: Peak list of the of the *G. glabra* root acetone extract chromatogram at 254 nm with a minimum area threshold of 0.5%. Table S7: Peak list of the of the *G. glabra* root 70% ethanol extract chromatogram at 254 nm with a minimum area threshold of 0.5%. Table S8: Peak list of the of the *R. palmatum* root extract chromatogram at 254 nm with a minimum area threshold of 0.5%. Table S9: Peak list of the of rhein chromatogram at 254 nm with a minimum area threshold of 0.5%.

## Abbreviations

BMD: broth microdilution; MIC: minimal inhibitory concentration.
