# Supplementary material for: Antimicrobial Activity of Eucalyptus globulus, Azadirachta indica, Glycyrrhiza glabra, Rheum palmatum Extracts and Rhein against Porphyromonas gingivalis
Source: Antibiotics (Basel). 2022 Jan 31;11(2):186. doi: 10.3390/antibiotics11020186 (PMC8868162; doi:10.3390/antibiotics11020186)
Supplement: Supplementary file 1 [file antibiotics-11-00186-s001.zip › antibiotics-1560834-supplementary.pdf]

Supplementary Materials

Table S1: Quality control ranges for *B. fragilis* and *B. thetaiotaomicron*. *MIC range according to CLSI [28],**MIC range according to MICRONAUT MERLIN Diagnostika.

**Antibiotics**	***B. fragilis*** **ATCC 25285 (mg/L)**	***B. thetaiotaomicron*** **ATCC 29741 (mg/L)**
Penicillin G	8-32*	8-32*
Amoxicillin/clavulancic acid	0.25/0.125 - 1/0.5*	0.25/0.125 - 1/0.5*
Piperacillin/tazobactam	≤4/4**	2/4 - 16/4*
Meropenem	0.03 - 0.25*	0.06 - 0.5*
Imipenem	0.03 - 0.25*	0.25 - 1*
Ertapenem	0.06 - 0,5*	0.25 - 2**
Metronidazole	0.25 - 2*	0.5 - 4*
Moxifloxacin	0.125 - 0.5*	1 - 8*
Clindamycin	0.5 - 2*	2 - 8*
Doxycycline	-	2 - 8*
Tigecycline	0.06 - 0.5*	0.25 - 1*

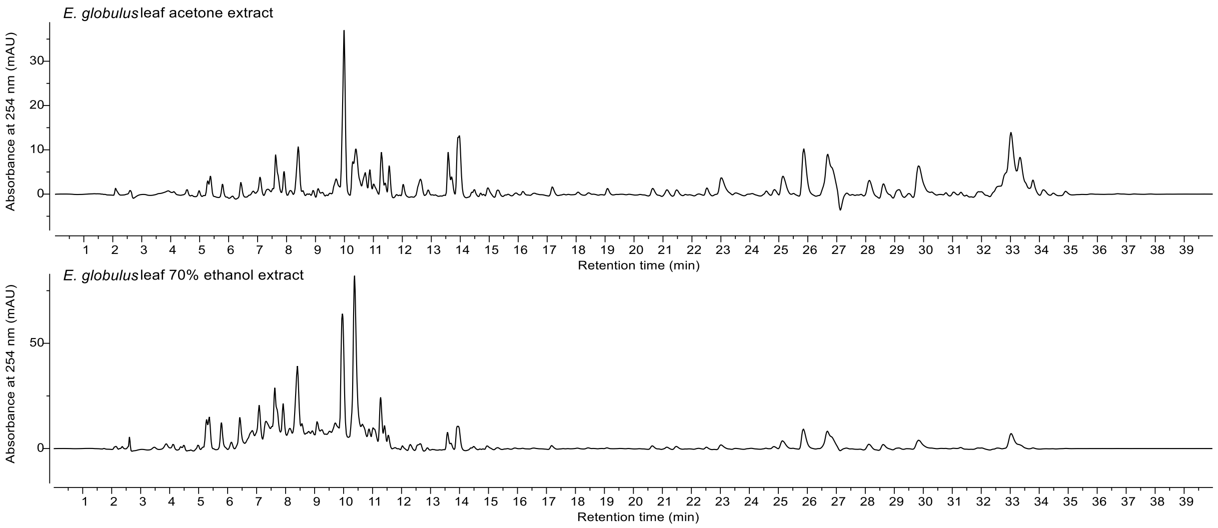

Figure S1: HPLC chromatograms (monitored at 254 nm) of *E. globulus* leaf acetone and 70% ethanol extracts.

Table S2: Peak list of the *E. globulus* leaf acetone extract chromatogram at 254 nm with a minimum area threshold of 0.5%.

Peak #	rt	Total Height %	Total Area %	Start time	End time	Peak #	rt	Total Height %	Total Area %	Start time	End time
**1**	5.277	1.24	0.65	5.184	5.317	**25**	14.956	0.72	0.71	14.862	15.169
**2**	5.371	1.64	1.02	5.317	5.497	**26**	17.175	0.78	0.70	17.049	17.389
**3**	5.790	1.11	0.67	5.671	5.917	**27**	20.648	0.69	0.81	20.495	20.961
**4**	6.424	1.29	0.82	6.324	6.570	**28**	21.135	0.58	0.66	20.968	21.341
**5**	7.084	1.44	1.02	6.924	7.210	**29**	21.475	0.54	0.57	21.341	21.695
**6**	7.630	2.88	2.16	7.550	7.817	**30**	22.514	0.66	0.59	22.401	22.701
**7**	7.917	1.84	1.02	7.823	8.023	**31**	23.014	1.38	2.08	22.848	23.401
**8**	8.403	3.77	3.02	8.237	8.523	**32**	25.141	1.56	2.36	25.001	25.700
**9**	9.716	1.22	1.42	9.563	9.850	**33**	25.860	3.82	4.65	25.700	26.160
**10**	9.983	13.33	10.10	9.850	10.130	**34**	26.687	3.85	9.76	26.400	27.114
**11**	10.283	2.77	1.37	10.190	10.316	**35**	27.340	1.29	2.29	27.127	27.474
**12**	10.396	3.82	4.50	10.316	10.590	**36**	27.534	1.10	1.17	27.474	27.634
**13**	10.716	1.93	1.99	10.590	10.803	**37**	27.694	1.02	0.84	27.634	27.753
**14**	10.876	2.19	1.47	10.803	10.956	**38**	27.840	1.05	1.24	27.753	27.933
**15**	11.010	1.04	1.05	10.956	11.183	**39**	28.127	2.17	4.80	27.933	28.460
**16**	11.276	3.66	2.60	11.183	11.370	**40**	28.613	1.93	3.91	28.460	28.953
**17**	11.410	1.17	0.67	11.370	11.476	**41**	29.133	1.09	2.04	28.953	29.340
**18**	11.543	2.60	1.59	11.476	11.690	**42**	29.480	0.46	0.62	29.340	29.640
**19**	12.029	1.00	0.64	11.956	12.223	**43**	29.826	2.48	4.14	29.640	30.240
**20**	12.623	1.39	1.60	12.396	12.776	**44**	33.019	3.57	4.55	32.846	33.213
**21**	13.583	3.56	2.25	13.483	13.663	**45**	33.333	2.04	2.51	33.213	33.599
**22**	13.702	1.58	1.04	13.663	13.816	**46**	33.779	0.90	0.78	33.659	33.972
**23**	13.922	4.85	2.21	13.816	13.936	**25**	14.956	0.72	0.71	14.862	15.169
**24**	13.969	5.00	3.36	13.936	14.162	**26**	17.175	0.78	0.70	17.049	17.389

Table S3: Peak list of the *E. globulus* leaf 70% ethanol extract chromatogram at 254 nm with a minimum area threshold of 0.5%.

Peak #	rt	Total Height %	Total Area %	Start time	End time	Peak #	rt	Total Height %	Total Area %	Start time	End time
**1**	5.273	2.92	1.82	5.167	5.313	19	10.879	1.40	1.10	10.806	10.952
**2**	5.367	3.22	2.32	5.313	5.493	20	11.019	1.62	2.06	10.952	11.186
**3**	5.787	2.66	1.83	5.673	5.920	21	11.279	4.98	3.75	11.186	11.366
**4**	6.420	3.08	2.68	6.273	6.593	22	11.412	2.19	1.47	11.366	11.492
**5**	6.846	1.46	2.40	6.593	6.926	23	11.546	1.31	0.89	11.492	11.666
**6**	7.086	3.95	3.91	6.926	7.206	24	12.652	0.77	1.04	12.459	12.772
**7**	7.320	2.15	3.15	7.206	7.473	25	13.585	1.50	0.88	13.505	13.672
**8**	7.526	1.52	0.80	7.473	7.546	26	13.919	2.42	1.22	13.819	13.939
**9**	7.626	5.42	5.94	7.546	7.826	27	13.965	2.45	1.80	13.939	14.158
**10**	7.913	3.61	2.67	7.826	8.033	28	23.017	0.48	0.75	22.870	23.390
**11**	8.146	0.93	0.96	8.033	8.233	29	25.143	0.83	1.37	24.997	25.697
**12**	8.406	7.20	7.48	8.233	8.526	30	25.863	2.05	2.80	25.703	26.170
**13**	8.559	1.17	0.71	8.526	8.666	31	26.690	1.89	4.66	26.410	27.130
**14**	9.086	1.24	0.78	9.013	9.186	32	28.129	0.61	0.92	27.943	28.463
**15**	9.713	1.11	1.44	9.579	9.853	33	28.616	0.59	0.90	28.469	28.949
**16**	9.959	12.57	11.91	9.853	10.132	34	29.843	0.89	1.69	29.643	30.256
**17**	10.379	16.78	17.91	10.206	10.599	35	33.029	1.35	1.97	32.835	33.282
**18**	10.652	1.68	2.00	10.599	10.806						

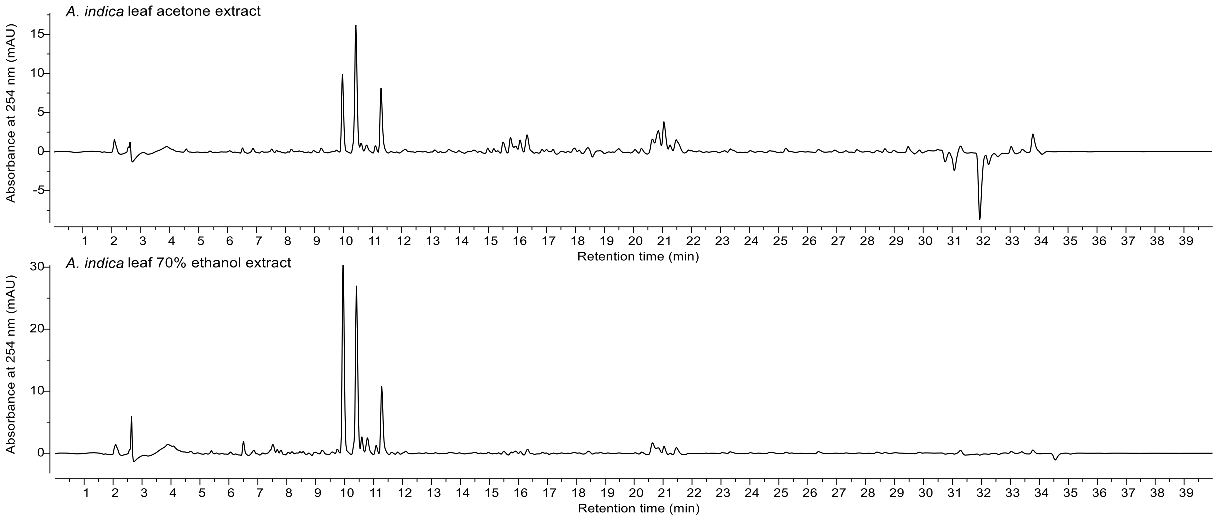

Figure S2: HPLC chromatograms (monitored at 254 nm) of *A. indica* leaf acetone and 70% ethanol extracts.

Table S4: Peak list the of the *A. indica* leaf acetone extract chromatogram at 254 nm with a minimum area threshold of 0.5%.

Peak #	rt	Total Height %	Total Area %	Start time	End time	Peak #	rt	Total Height %	Total Area %	Start time	End time
**1**	2.092	1.93	1.04	1.985	2.272	**18**	21.475	1.88	1.89	21.369	21.762
**2**	3.078	1.11	2.02	2.705	3.218	**19**	26.388	0.50	0.78	26.268	26.848
**3**	3.891	2.32	9.61	3.218	4.471	**20**	26.948	0.55	0.74	26.848	27.248
**4**	4.565	1.00	0.91	4.471	4.831	**21**	27.341	0.52	0.75	27.248	27.627
**5**	9.957	10.79	4.60	9.864	10.090	**22**	27.714	0.76	1.70	27.627	28.281
**6**	10.424	16.72	7.60	10.330	10.550	**23**	28.407	0.73	0.95	28.281	28.594
**7**	11.290	8.73	4.06	11.197	11.430	**24**	28.681	1.02	1.05	28.594	28.907
**8**	15.503	1.60	0.94	15.410	15.656	**25**	28.987	0.75	1.20	28.907	29.381
**9**	15.763	2.17	1.30	15.656	15.876	**26**	29.481	1.22	1.01	29.381	29.707
**10**	15.936	0.93	0.55	15.876	16.016	**27**	30.434	0.92	0.79	30.120	30.440
**11**	16.089	1.75	0.91	16.016	16.216	**28**	30.640	1.44	1.76	30.440	30.754
**12**	16.336	2.36	1.40	16.216	16.476	**29**	30.907	1.94	1.90	30.760	31.067
**13**	18.436	1.14	1.05	18.256	18.582	**30**	31.840	8.30	24.07	31.080	31.953
**14**	20.655	2.09	1.44	20.489	20.735	**31**	32.120	6.98	7.86	31.953	32.260
**15**	20.862	3.24	2.78	20.735	20.969	**32**	32.347	4.40	6.17	32.260	32.587
**16**	21.055	4.46	2.92	20.969	21.202	**33**	32.587	1.93	1.40	32.587	32.840
**17**	21.262	1.18	0.70	21.202	21.369	**34**	33.786	2.67	2.12	33.653	34.093

Table S5: Peak list of the *A. indica* leaf 70% ethanol extract chromatogram at 254 nm with a minimum area threshold of 0.5%.

Peak #	rt	Total Height %	Total Area %	Start time	End time	Peak #	rt	Total Height %	Total Area %	Start time	End time
**1**	2.087	1.67	2.16	1.974	2.261	**14**	10.593	2.17	1.52	10.533	10.679
**2**	2.634	6.01	2.65	2.594	2.687	**15**	10.786	2.46	2.68	10.686	10.993
**3**	3.061	0.76	2.60	2.721	3.207	**16**	11.086	1.43	1.18	11.006	11.186
**4**	3.887	1.88	9.80	3.207	4.054	**17**	11.279	10.53	9.06	11.193	11.406
**5**	4.094	1.47	2.17	4.054	4.254	**18**	16.318	0.83	0.91	16.198	16.438
**6**	4.254	0.65	0.91	4.254	4.554	**19**	20.638	1.92	2.82	20.478	20.751
**7**	6.507	2.23	1.73	6.427	6.640	**20**	20.838	1.10	1.73	20.751	20.951
**8**	6.860	0.73	0.79	6.687	6.967	**21**	21.031	1.34	1.46	20.951	21.178
**9**	7.520	1.31	1.54	7.346	7.613	**22**	21.457	1.16	1.71	21.337	21.724
**10**	9.000	0.42	0.61	8.873	9.140	**23**	31.276	0.70	1.06	31.063	31.429
**11**	9.219	0.57	0.62	9.140	9.353	**24**	33.775	0.64	0.78	33.649	33.989
**12**	9.946	31.76	25.72	9.853	10.073	**25**	34.735	0.67	2.15	34.555	35.075
**13**	10.406	25.59	21.64	10.319	10.533						

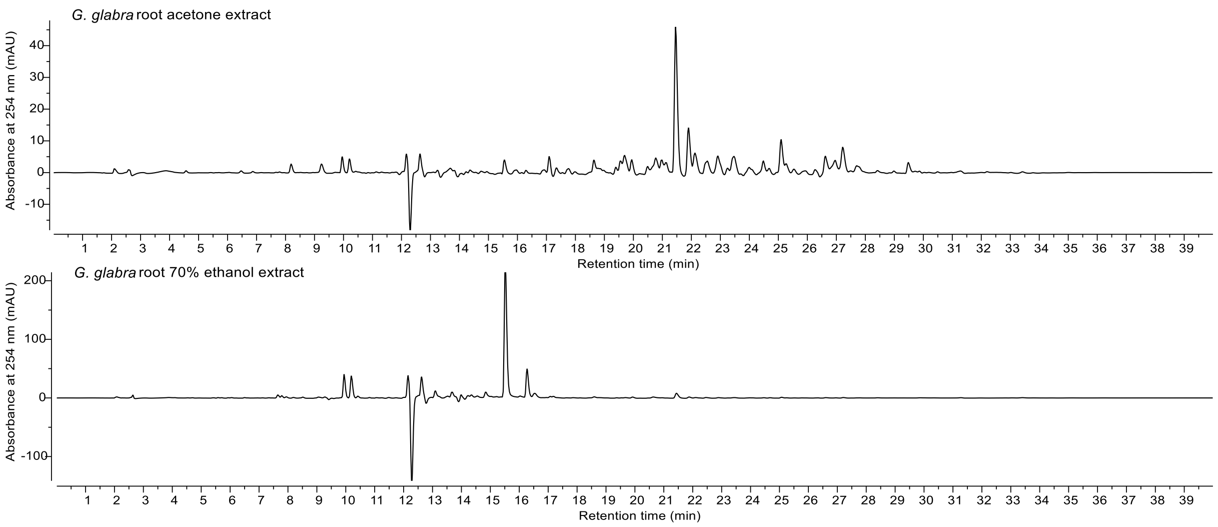

Figure S3: HPLC chromatograms (monitored at 254 nm) of *G. glabra* root acetone and 70% ethanol extracts.

Table S6: Peak list of the of the *G. glabra* root acetone extract chromatogram at 254 nm with a minimum area threshold of 0.5%.

Peak #	rt	Total Height %	Total Area %	Start time	End time	Peak #	rt	Total Height %	Total Area %	Start time	End time
**1**	12.070	2.12	0.88	11.930	12.076	**20**	16.289	1.47	1.55	16.216	16.436
**2**	12.183	4.81	3.86	12.076	12.296	**21**	16.436	1.09	1.03	16.436	16.622
**3**	12.636	6.07	11.98	12.296	12.796	**22**	16.622	0.91	0.70	16.622	16.776
**4**	12.903	4.30	4.77	12.796	13.003	**23**	16.902	0.95	1.09	16.776	17.009
**5**	13.003	4.22	3.64	13.003	13.163	**24**	17.095	1.88	1.22	17.009	17.229
**6**	13.236	4.20	4.05	13.163	13.349	**25**	17.342	0.74	0.66	17.229	17.629
**7**	13.676	3.97	8.98	13.349	13.783	**26**	19.695	0.88	0.58	19.615	19.848
**8**	13.816	3.67	2.99	13.783	13.943	**27**	19.942	1.04	0.59	19.855	20.088
**9**	14.023	3.22	3.44	13.943	14.143	**28**	20.768	0.88	0.65	20.575	20.895
**10**	14.223	3.20	2.61	14.143	14.296	**29**	21.455	11.81	7.78	21.335	21.668
**11**	14.363	3.22	3.67	14.296	14.516	**30**	21.901	3.85	2.63	21.768	22.035
**12**	14.516	2.87	2.14	14.516	14.656	**31**	22.121	1.72	1.41	22.035	22.328
**13**	14.743	2.78	3.23	14.656	14.876	**32**	22.548	1.00	0.98	22.381	22.748
**14**	14.936	2.58	3.02	14.876	15.102	**33**	22.915	1.45	1.33	22.748	23.134
**15**	15.102	2.26	1.43	15.102	15.222	**34**	23.474	1.29	1.20	23.321	23.654
**16**	15.316	2.09	2.77	15.222	15.469	**35**	25.094	2.39	1.35	24.988	25.214
**17**	15.549	2.98	3.58	15.469	15.769	**36**	26.621	1.62	1.34	26.434	26.767
**18**	15.896	1.81	2.92	15.769	16.089	**37**	26.954	1.14	1.31	26.767	27.087
**19**	16.136	1.42	0.95	16.089	16.216	**38**	27.220	2.09	1.70	27.087	27.420

Table S7: Peak list of the of the *G. glabra* root 70% ethanol extract chromatogram at 254 nm with a minimum area threshold of 0.5%.

Peak #	rt	Total Height %	Total Area %	Start time	End time	Peak #	rt	Total Height %	Total Area %	Start time	End time
**1**	9.944	4.47	2.62	9.851	10.091	**8**	13.677	1.24	1.33	13.590	13.890
**2**	10.191	4.21	2.69	10.097	10.344	**9**	13.983	1.17	0.78	13.910	14.117
**3**	12.190	12.39	10.74	12.057	12.270	**10**	14.836	0.99	0.66	14.743	14.990
**4**	12.410	14.68	20.32	12.284	12.537	**11**	15.516	25.28	17.42	15.416	15.690
**5**	12.617	15.42	20.37	12.537	12.777	**12**	16.270	5.38	3.55	16.176	16.430
**6**	12.777	7.80	11.06	12.777	13.010	**13**	16.530	0.70	0.66	16.430	16.723
**7**	13.083	5.32	6.98	13.010	13.337	**14**	21.435	0.95	0.81	21.315	21.642

Table S8: Peak list of the of the *R. palmatum* root extract chromatogram at 254 nm with a minimum area threshold of 0.5%. The HPLC chromatogram (monitored at 254 nm), the respective total ion current (TIC) ESI-MS (–) and the extracted ESI-MS (–) spectrum of the *R. palmatum* root sample are depicted in the main article (Figure 2).

Peak #	rt	Total Height %	Total Area %	Start time	End time	Peak #	rt	Total Height %	Total Area %	Start time	End time
1	4.898	734	1.08	0.97	4.778	16	12.117	1817	1.52	1.12	12.05
2	5.558	833	0.92	0.87	5.431	17	12.216	1832	1.73	2	12.163
3	8.277	1241	2.63	2.48	8.191	18	13.956	2093	8.35	9.27	13.816
4	8.937	1340	1.08	1.33	8.81	19	14.176	2126	2.39	1.95	14.103
5	9.37	1405	1.39	3.57	9.204	20	14.296	2144	7.62	7.67	14.223
6	9.757	1463	1.27	1.72	9.617	21	15.103	2265	2.79	2.54	15.009
7	10.137	1520	13.42	11.55	10.05	22	15.423	2313	2.78	3.85	15.323
8	10.29	1543	24.17	20.9	10.223	23	16.356	2453	0.66	0.76	16.262
9	10.457	1568	1.86	1.93	10.403	24	17.502	2625	3.01	3.06	17.402
10	10.623	1593	2.66	1.93	10.557	25	18.635	2795	0.73	0.88	18.515
11	10.723	1608	5.63	5.72	10.657	26	19.022	2853	2.25	2.78	18.895
12	10.883	1632	0.74	0.67	10.837	27	20.948	3142	0.66	1.01	20.802
13	11.037	1655	0.91	1.47	10.963	28	22.808	3421	0.5	0.64	22.675
14	11.443	1716	1.49	1.45	11.37	29	25.781	3867	1.67	2.36	25.634
15	11.597	1739	4.1	3.55	11.53						

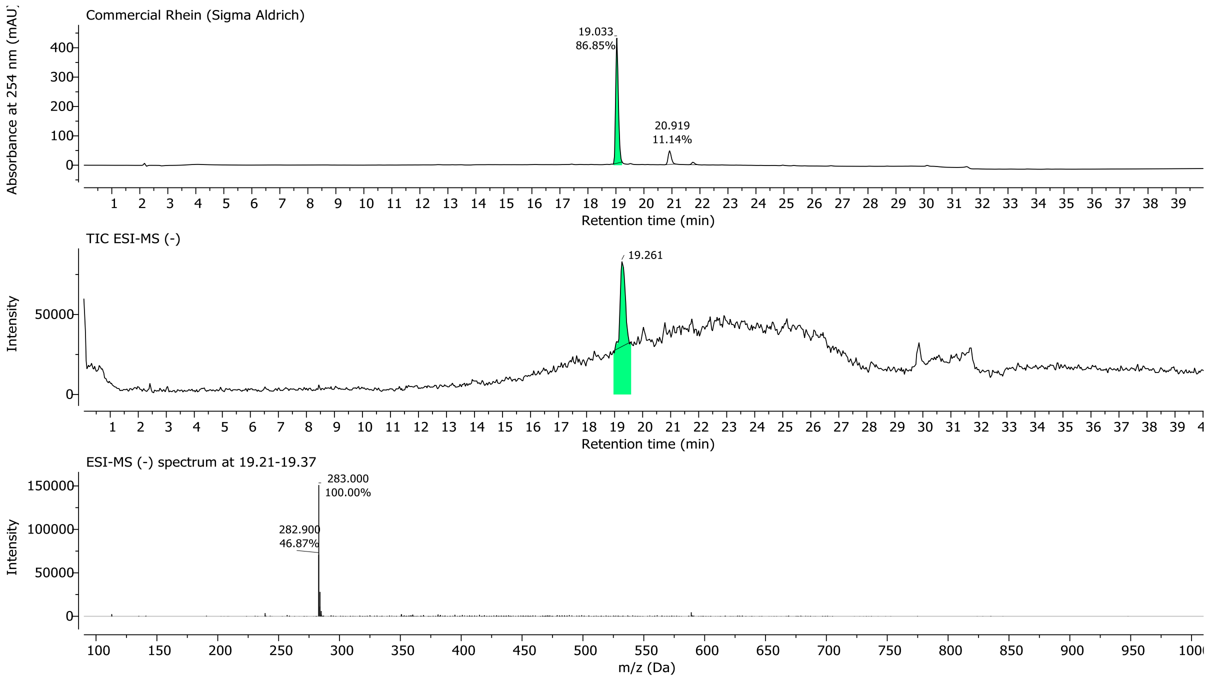

Figure S4: HPLC chromatogram (monitored at 254 nm), respective total ion current (TIC) ESI-MS (–) and extracted ESI-MS (–) spectrum at 19.21-19.37 min (green, rhein) of a commercial rhein sample (Sigma Aldrich).

Table S9: Peak list of the of rhein chromatogram at 254 nm with a minimum area threshold of 0.5%.

Peak #	rt	Total Height %	Total Area %	Start time	End time
1	19.033	90.22	88.64	18.846	19.313
2	20.919	9.78	11.36	20.633	21.193
